# Application of α-aminoisobutyric acid and β-aminoisobutyric acid inhibits pericarp browning of harvested longan fruit

**DOI:** 10.1186/s13065-015-0124-1

**Published:** 2015-10-06

**Authors:** Hui Wang, Wei Zhi, Hongxia Qu, Hetong Lin, Yueming Jiang

**Affiliations:** Key Laboratory of Plant Resources Conservation and Sustainable Utilization, South China Botanical Garden, Chinese Academy of Sciences, 510650 Guangzhou, China; Institute of Postharvest Technology of Agricultural Products, College of Food Science, Fujian Agriculture and Forestry University, 350002 Fuzhou, China

**Keywords:** Longan, Storage, Pericarp browning, High-performance liquid chromatography, Phenolic compounds

## Abstract

**Background:**

Pericarp browning is a critical problem resulting in reduced commercial value and shelf life of longan fruit.

**Results:**

Two non-protein amino acids, α-aminoisobutyric acid (AIB) and β-aminoisobutyric acid (BAIB) at 100 and 1 mM were applied to longan fruit prior to storage for up to 8 days at 25 °C respectively. Contents of the major five phenolics (gallic acid, catechin, corilagin, epicatechin and gallocatechin gallate) were assayed by high-performance liquid chromatography (HPLC). Physiological properties related to pericarp browning of longan fruit were investigated during storage. Respiration rate, membrane permeability, malondialdehyde (MDA) content, and activities of polyphenol oxidase (PPO) and peroxidase (POD) were down-regulated by AIB or BAIB treatments, with significantly lower pericarp browning index and higher proportion of edible fruit than the control. Moreover, exogenous application of AIB and BAIB maintained higher contents of catechin, corilagin, epicatechin and gallocatechin gallate, but lower content of gallic acid compared to the control in the pericarp of longan fruit during storage, which was associated with the oxidation of browning substrate.

**Conclusions:**

Pericarp browning was inhibited and storage life of longan fruit was extended effectively by AIB and BAIB treatments with AIB treatment being more significant than BAIB. The findings may provide a new strategy for controlling pericarp browning of harvested longan fruit.

## Background

Longan (*Dimocarpus longan* Lour.), a member of the *Sapindaceae* family, is a subtropical fruit with high commercial value for its flavor and nutrition. Longan fruit pericarp turns brown rapidly after 2 or 3 days of harvest [1]. Longan fruit pericarp contains large amounts of phenolics, thereby exhibiting strong antioxidant activities [2]. It was reported that pericarp browning was mainly due to the formation of brown substances caused by oxidation of phenolics by polyphenol oxidase (PPO) and peroxidase (POD) [3]. Thus, the inhibition of oxidation of browning substrate could be an alternative for controlling pericarp browning of harvested longan fruit.

α-Aminoisobutyric acid (AIB), a non-protein amino acid, is an analogue of 1-aminocyclopropane-1-carboxylic acid (ACC) as the immediate precursor of ethylene in higher plants [4]. AIB inhibits ethylene production by acting as a competitive inhibitor of conversion of ACC to ethylene. AIB has been reported to delay carnation wilting and extend the vase life of cut flowers [5, 6]. Aminobutyric acid derivative, such as β-aminobutyric acid (BABA), an inducer of disease resistance in plants possesses a large spectrum of activity in inducing resistance against disease in peas, potato, tomato, tobacco, apple, grapevine, cucumber, and other plants [7, 8]. It was found that BABA could enhance the defense resistance of potato to phytopathogens such as *Phytophthora infestans* and *Fusarium solani* [9]. While γ-aminobutyric acid (GABA) as another derivative can accumulate proline, reduce chilling injury and activate the defense response of peach fruit during long-term cold storage [10, 11]. As a structural isomers of AIB, β-aminoisobutyric acid (BAIB) has a key metabolic role in enhancing fatty acid oxidation by converting the cells of white adipose tissue into brown fat and turning off the energy stores, which may help to prevent diet-induced obesity and related metabolic disorders [12]. Unfortunately, information is extremely limited on roles of BAIB in harvested horticultural crops. Thus, it can be expected AIB and BAIB as postharvest alternatives for longan fruit by inducing defense activity and delaying senescence.

In this study, AIB and BAIB were applied on harvested longan fruit before storage. Changes of pericarp browning, fruit decay, respiration rate, membrane permeability, PPO and POD activities and total phenolic contents in fruit pericarp were evaluated during storage, while the major phenolic compounds were identified. The roles of α-aminoisobutyric acid and β-aminoisobutyric acid treatments in delaying pericarp browning of harvested longan fruit were proposed.

## Results

### Pericarp browning, proportion of edible fruit and respiration intensity

**Fig. 1:**
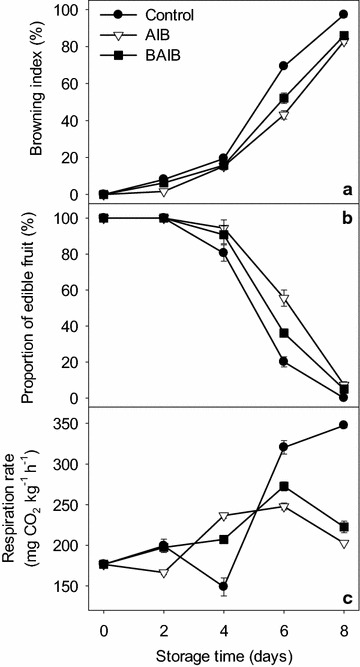
Browning index (**a**), proportion of edible fruit (**b**) and respiration rate (**c**) in longan fruit during storage at 25 °C. Longan fruit was treated with water (control), α-aminoisobutyric acid (AIB) or β-aminoisobutyric acid (BAIB). Values represent the mean ± SE of three to five replicates

Browning index of longan fruit increased gradually within the first 2 days and thereafter increased rapidly as storage time progressed (Fig. 1a). α-Aminoisobutyric acid (ΑΙΒ) and β-aminoisobutyric acid (BAIB) treatments delayed significantly the increase in pericarp browning index (Fig. 1a). The browning indices were 69.32 and 97.22 % on the 6th and 8th day of storage in control fruit, but only 43.06 and 82.89 % in the ΑΙΒ-treated fruit, decreased by 37.89 and 14.74 %, respectively. The browning indices of the BAIB-treated fruit were 52.08 and 85.92 %, decreased by 24.86 and 11.62 %, respectively.

The proportion of edible fruit decreased rapidly after 4 days of storage. Exogenous ΑΙΒ and BAIB treatments maintained higher proportion of edible fruit compared with the control fruit (Fig. 1b). It was suggested that application of ΑΙΒ and BAIB effectively delayed pericarp browning, inhibited deterioration, and thus maintained the commercial value of longan fruit after harvest.

Respiration rate of longan fruit increased slowly after harvest, then decreased after 2 days, but increased rapidly after 4 days of storage (Fig. 1c). ΑΙΒ and BAIB treatments postponed the peak of the respiration rate, which appeared on the 6th day of storage in ΑΙΒ and BAIB-treated fruits. Exogenous ΑΙΒ and BAIB applications down-regulated significantly the respiration intensity at the end of storage, which were decreased by 22.68 and 14.95 % on the 6th day, and 41.62 and 35.91 % on the 8th day of storage, respectively.

### Membrane permeability and MDA content

**Fig. 2:**
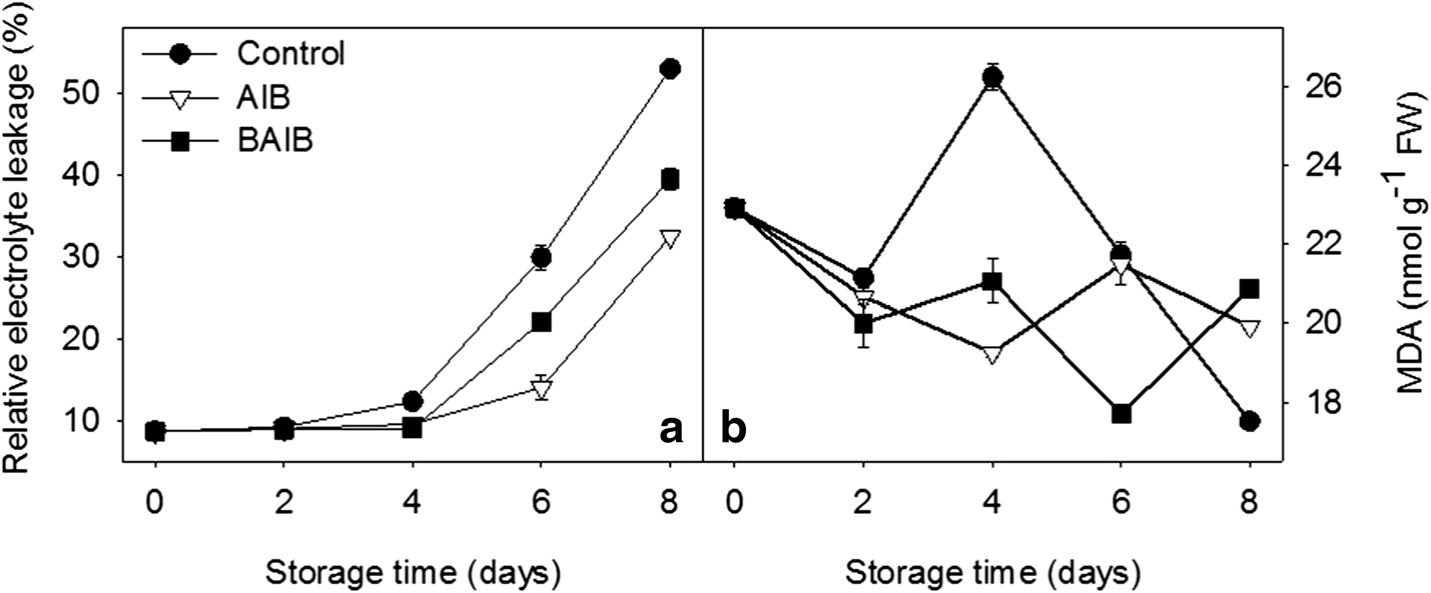
Relative electrolyte leakage (**a**) and MDA content (**b**) in pericarp of longan fruit during storage at 25 °C. Longan fruit was treated with water (control), α-aminoisobutyric acid (AIB) or β-aminoisobutyric acid (BAIB). *MDA* malondialdehyde, *FW* fresh weight. Values represent the mean ± SE of three replicates

Membrane permeability reflects senescence and deterioration of plant tissues and is expressed by relative electric conductivity. As shown in Fig. 2a, membrane permeability of longan pericarp increased slightly within the first 4 days and then increased rapidly. The electric conductivities in the ΑΙΒ and BAIB-treated fruit were 32.49 and 39.44 % after 8 days of storage, respectively, while the control fruit had electric conductivity of 52.94 %. ΑΙΒ and BAIB application inhibited significantly the increase of relative electric conductivity (by 38.64 and 25.50 % respectively) at the end of storage.

Malondialdehyde (MDA), a low molecular weight end-product was supposed to be resulted from oxidative damage of membrane lipids [13]. As shown in Fig. 2b, MDA content decreased within the first 2 days and peaked remarkably at the 4th day of storage in the control fruit. MDA content declined in control between 4 and 8 days, which was in contrast with the result on membrane permeability. A possible explanation about MDA decrease in control between 4 and 8 days may be due to the degradation of MDA itself or may be resulted from being utilized by the pathogens. Both ΑΙΒ and BAIB treatments inhibited significantly the fluctuation. Moreover, a lower MDA contents were observed in the fruit treated with ΑΙΒ or BAIB than that in the control.

### PPO and POD activities

**Fig. 3:**
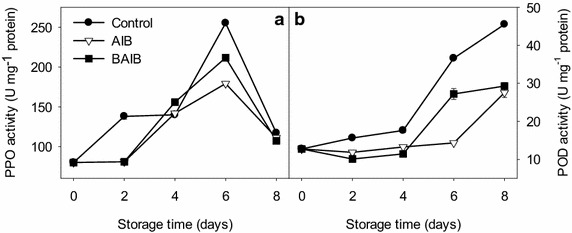
PPO (**a**) and POD (**b**) activities in pericarp of longan fruit during storage at 25 °C. Longan fruit was treated with water (control), α-aminoisobutyric acid (AIB) or β-aminoisobutyric acid (BAIB). *PPO* polyphenol oxidase, *POD* peroxidase. Values represent the mean ± SE of three replicates

Numerous research works have indicated that PPO and POD were involved in the oxidation of phenolic compounds and consequently resulted in pericarp browning [3]. In this study, PPO activity increased within the first 6 days of storage in the pericarp of longan fruit and then decreased (Fig. 3a). Treatments with ΑΙΒ and BAIB inhibited the increase of PPO activity and down-regulated it by 29.75 and 17.00 % at the 6th day of storage, respectively.

POD activity in longan fruit pericarp tissues increased steadily and thereafter increased significantly at the last 4 days. Application of AIB and BAIB reduced the increase in POD activity by 39.52 and 35.67 % respectively by the end of the storage (Fig. 3b).

### Total phenolics and flavonoids

**Fig. 4:**
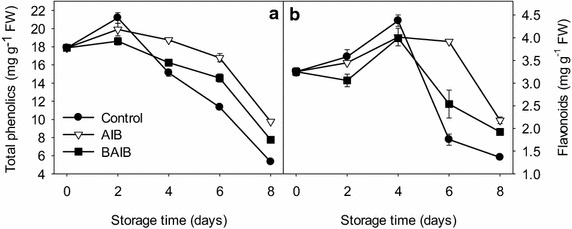
Total phenolics (**a**) and flavonoids (**b**) in longan fruit during storage at 25 °C. Longan fruit was treated with water (control), α-aminoisobutyric acid (AIB) or β-aminoisobutyric acid (BAIB). *FW* fresh weight. Values represent the mean ± SE of three replicates

The concentration of total phenolics increased significantly within the first 2 days of storage before declining as storage progressed, and exogenous AIB and BAIB treatments blocked these changes (Fig. 4a). The concentrations of total phenolics were 9.76 and 7.76 mg g^−1^ FW in the AIB and BAIB-treated fruits, respectively, while the concentration of the control fruit was 5.33 mg g^−1^ FW by the end of storage.

Flavonoids content increased within the first 4 days of storage and declined thereafter significantly, and AIB and BAIB treatments slowed these changes (Fig. 4b). Flavonoids content in the AIB and BAIB-treated fruit was 60.00 and 41.14 %, respectively, higher than that in the control fruit by the end of storage.

### Phenolic components

**Fig. 5:**
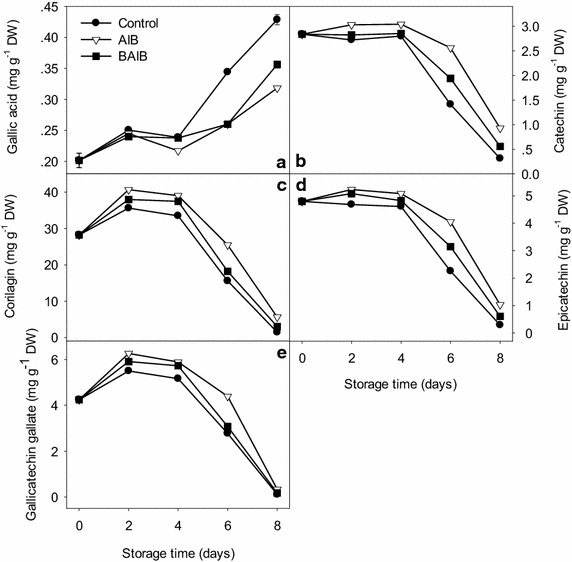
Contents of gallic acid (**a**), catechin (**b**), corilagin (**c**), epicatechin (**d**) and gallocatechin gallate (**e**) in pericarp of longan fruit during storage at 25 °C. Longan fruit was treated with water (control), α-aminoisobutyric acid (AIB) or β-aminoisobutyric acid (BAIB). *DW* dry weight. Values represent the mean ± SE of three replicates

**Fig. 6:**
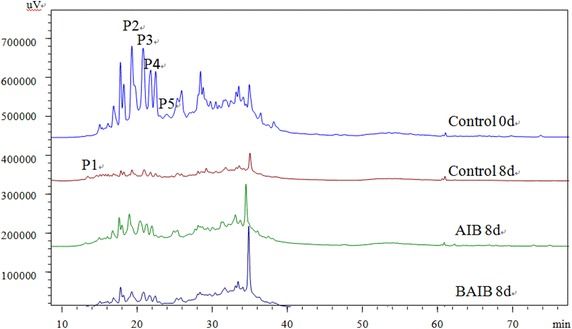
HPLC chromatogram of phenolic components from longan pericarp before storage (0 day) and after stored for 8 days at 25 °C. *P1* gallic acid, *P2* catechin, *P3* corilagin, *P4* epicatechin, *P5* gallocatechin gallate

Longan fruit contains rich polyphenolic compounds [14]. Five phenolics were identified from longan pericarp in the present study. Among them, corilagin was the most abundant (35.60 mg g^−1^ DW at the 2nd day of storage), followed by epicatechin, gallocatechin gallate, catechin and gallic acid. HPLC analysis showed that the content of gallic acid peaked after 2 days of storage. Exogenous AIB and BAIB treatments significantly inhibited the rise of gallic acid content. Gallic acid was reduced by 25.75 and 16.86 % in the AIB and BAIB-treated fruit compared with that in the control fruit by the end of the storage (Figs. 5a, 6). Concentrations of catechin and epicatechin remained relatively stable within the first 4 days and declined significantly thereafter (Fig. 5b, d). AIB and BAIB treatments maintained higher levels of catechin and epicatechin after 8 days of storage than the control (Fig. 6). The line trend of gallocatechin gallate was similar with that of corilagin in longan fruit during storage. Exogenous AIB and BAIB treatments maintained higher levels of corilagin and gallocatechin gallate than the control during storage (Fig. 5c, e).

## Discussion

Longan fruit turns brown and deteriorates within 2–4 days at ambient temperature after harvest. Proper treatment can prolong shelf life to 7–9 days at room temperature. Ethylene plays an important role in ripening and senescence of horticultural products including fruits, flowers and vegetables. α-Aminoisobutyric acid as an analogue of 1-aminocyclopropane-1-carboxylic acid could inhibit ethylene production, and extend the vase life of cut flowers [4, 5]. Longan is a non-climacteric fruit and produces relatively low level of ethylene after harvest compared with climacteric fruits. It was reported that two ethylene-responsive factor-like genes (DIRRF1 and DIERF2) were involved in regulating longan fruit senescence [15]. The ethylene receptor inhibitors were effective in reducing internal browning of pineapple, apricot and plum. Moreover, anti-ethylene (1-methylcyclopropene and CoCl_2_) treatments could reduce postharvest disease and browning incidence of litchi fruit, a member of the *Sapindaceae* family [16]. Thus, the effect of exogenous AIB treatment on delaying pericarp browning of longan fruit might be related to its roles on ethylene metabolism in the present study, although there was no significant difference in ethylene level between the treatments and the control (data not shown).

Previous studies have shown that aminobutyric acid and its derivatives were effective in inducing resistance against pathogens in many fruit [7, 8]. β-Aminobutyric acid was also effective in inducing resistance against oomycete pathogens, bacteria, fungi and virus [7, 8, 17]. Furthermore, application of their derivative γ-aminobutyric acid (GABA) reduced chilling injury and activated the defense response of peach fruit [10, 11]. In this study, AIB and BAIB application remarkably delayed pericarp browning, extended storage time, maintained higher proportion of edible fruit and decreased respiration rate (Fig. 1a–c). All of the effects give insight to understanding the roles of AIB and BAIB, and to develop potential applications of these aminobutyric acid derivatives in harvested horticultural products.

Postharvest browning of longan fruit is thought to be associated with oxidation of phenolics to quinines and then condense tannins to brown polymers by polyphenol oxidase (PPO) and peroxidase (POD) enzymes [3, 18]. In this investigation, application of AIB and BAIB effectively reduced the levels of the PPO and POD activity developed during storage (Fig. 3a, b) and maintained high levels of phenolics and flavonoids (Fig. 4a, b) during storage. These results supported the evidence that inhibiting of PPO and POD activities may prevent pericarp browning of longan fruit. In addition, deterioration in membrane integrity may result in loss of compartmentation of enzymes and substrates, consequently leading to pericarp browning. Treatments of AIB and BAIB effectively maintained membrane integrity and reduced MDA content (Fig. 2a, b). Taken together, these data can partially elucidate the effects of the inhibition of pericarp browning of harvested longan fruit by application of α-aminoisobutyric acid and β-aminoisobutyric acid.

Longan pericarp contains abundant phenolic compounds and flavonoids. High contents of phenolic compounds and flavonoids delay the pericarp browning process [3, 19]. Furthermore, phenolic compounds and flavonoids exhibited antibacterial, antiviral, antioxidant, anti-inflammatory and anti-carcinogenic properties [20, 21]. Different phenolic components, such as gallic acid, corilagin and epicatechin have been identified from longan fruit in the previous studies [14]. In the present study, five phenolic compounds, gallic acid, corilagin, catechin, epicatechin and gallocatechin gallate were isolated and analyzed during storage. The contents of these phenolics showed dynamic changes. Gallic acid increased during storage whereas other four phenolic compounds declined (Figs. 5, 6). The increase of gallic acid may be due to degradation of other ellagitannins or gallotannins by nonenzymatic or enzymatic hydrolysis, releasing free forms of gallic acid [14]. AIB and BAIB application effectively inhibited the increase of gallic acid content and decrease of corilagin, catechin, epicatechin and gallocatechin gallate contents in longan pericarp during storage. It was suggested that oxidation of phenolics was reduced by AIB and BAIB treatments and PPO and POD activities were inhibited, thus resulting in reduced browning of longan pericarp and extended shelf life.

## Conclusions

In conclusion, AIB and BAIB treatments effectively inhibited pericarp browning and extended storage life of harvested longan fruit, which may be resulted from decreasing respiration intensity, maintaining membrane integrity, and inhibiting PPO and POD activities and oxidation of phenolic compounds. This research provides a promising method for delaying pericarp browning and extending shelf life of longan fruit by exogenous application of AIB and BAIB. However, further studies are necessary to clarify the complex molecular networks regulated by AIB and BAIB on pericarp browning and senescence of harvested longan fruit during storage.

## Methods

### Fruit materials and treatments

Fruit of longan (*Dimocarpus longan* Lour. cv. Shixia) at commercial maturation stage were freshly harvested from an orchard in Guangzhou, China. They were selected for uniformity of shape, size, colour, and for freedom from blemish and disease. The fruits were surface-sterilized by dipping for 3 min in 0.05 % Sportak solution, air dried and divided into three groups (240 fruits per group). Then they were vacuum-infiltrated (75 kPa) with sterile distilled water (control), 100 mM α- aminoisobutyric acid and 1 mM β-aminoisobutyric acid for 3 min, respectively. These fruits were air-dried, packed with 0.015 mm thick polyethylene bags (20 fruits per bag, 12 bags per treatment), and then stored at 25 °C and 85–90 % relative humidity for up to 8 days. Sixty freshly harvested fruits were sampled before treatment and used for 0 day assessments. After 2, 4, 6 and 8 days of storage, three bags of fruit per treatment were used for measurements. Respiration rate, browning index and the proportion of edible fruit were determined using fresh sample, the remaining pericarp was stored under −20 °C for enzyme activity and phenolic component determinations. Each parameter was tested using the following methods with three to five replicates per measurement.

### Pericarp browning and the proportion of edible fruit

Fruit browning was assessed by measuring the extent of the total browned area of inner pericarp of 60 fruits, using the following scale: 0, no browning (excellent quality); (1) slight browning; (2) <1⁄4 browning; (3) 1⁄4–1⁄2 browning; (4) >1⁄2 browning (poor quality). A browning index (%) was calculated as: ∑ (browning scale × proportion of corresponding fruit within each class).

Disease development was monitored using 60 randomly selected fruits and recorded as the ratio of mean proportion (%) of the fruit surface with fungal growth to the total number of fruit. The rating scale described above for browning was also applied for disease severity. The proportion of edible fruit was calculated as the percentage of the total number of fruit exhibiting a disease scale value ≤1.

### Respiration rate

Respiratory rate of longan fruit was measured by infrared CO_2_ analysis with a Li-6262 CO_2_/H_2_O analyzer (LI-COR, Inc., Lincoln, NE, USA). Fifteen longan fruits were sealed in a 2.6 L easy-lock food container and the CO_2_ concentration in the container were monitored for 5 min by passing the air stream through the analyzer. Respiration rate was expressed as carbon dioxide released per kilogram per hour of fresh weight (mg CO_2_ kg^−1^ h^−1^). The analysis was carried out using five replicate fruit samples.

### Membrane permeability and malondialdehyde (MDA)

Membrane permeability was expressed as the relative electrolyte leakage. Twenty pericarp discs were removed with a cork borer (10 mm in diameter) from the equatorial region of 30 individual fruit, washed three times in deionized water, dried with filter paper, incubated in 20 mL sterile distilled water at 25 °C and shaken for 30 min. Initial electrolyte leakage was determined with a DDS-11A conductivity meter (Shanghai Scientific Instruments, Shanghai, China). Total electrolyte leakage was then determined after boiling for 20 min and then cooled to 25 °C. The relative leakage was expressed as a percentage of the initial electrolyte to the total electrolyte.

MDA content was measured according to the method of Zhang et al. [22]. Pericarp tissues were ground in liquid nitrogen and extracted in 15 mL of 5 % (w/v) trichloroacetic acid (TCA) before centrifuge at 8000*g* for 10 min at 4 °C. The reaction mixture consisting of 1 mL supernatant and 3 mL of 0.5 % (w/v) thiobarbituric acid (TBA) containing 10 % TCA was incubated in a boiling water bath for 20 min, then cooled quickly in an ice bath and finally centrifuged at 8000*g* for 10 min at 4 °C. Absorbance of the supernatant was recorded at 532 nm. MDA content was calculated using an extinction coefficient of 155 mM^−1^ cm^−1^, and then expressed as mmol g^−1^ FW.

### PPO and POD activities

Pericarp tissue (2 g) were ground in liquid nitrogen and then extracted with 8 mL of 0.2 M phosphate buffer (pH 6.8) and 0.2 g of polyvinylpyrrolidone at 4 °C. The clear supernatant was collected as enzyme extracts after centrifugation at 15,000*g* for 15 min at 4 °C. PPO activity was measured according to the method of Duan et al. [3] by measuring the oxidation of 4-methylcatechol. The reaction mixture (3 mL) consisted of 50 μL enzyme solution, 0.45 mL of 0.2 M sodium phosphate buffer (pH 6.8) and 2.5 mL of 50 mM 4-methylcatechol. One unit of enzyme activity was defined as the amount that caused a change of 0.001 in absorbance at 398 nm per minute. For POD activity, guaiacol was used as the enzyme substrate by the method of Duan et al. [3]. The reaction mixture (3 mL) consisted of 15 μL enzyme solution, 2.925 mL of 0.2 M sodium phosphate buffer (pH 6.8), 30 μL of 0.046 % H_2_O_2_, and 30 μL of 4 % guaiacol. One unit of enzyme activity was defined as the amount that caused a change of 0.001 in absorbance at 410 nm per minute. Protein content was determined with bovine serum as the standard.

### Total phenolics and flavonoids

The phenolic contents in fruit pericarp were determined by the Folin–Ciocalteu method. The phenolic content was expressed as gallic acid equivalents in milligrams on fresh weight basis. Flavonoids content was measured referring to Yao et al. [23]. One milliliter of filtrate, 3.5 mL methanol and 0.5 mL of 50 g L^−1^ sodium nitrite were added to 100 mg L^−1^ aluminum nitrate solution, and incubated for 6 min before 4 mL of 40 g L^−1^ sodium hydroxide was added and incubated for an additional 15 min. The total flavonoids were expressed as the lutin equivalents in milligrams on a fresh weight (FW) basis.

### Phenolic components

Pericarp tissue (5 g) were ultrasonically (240 W) extracted with 50 mL of 80 % ethanol solution for 30 min at 30 °C and then filtered. The filtrate was extracted twice. The filtrate was combined and diluted with 80 % ethanol solution to 200 mL for high-performance liquid chromatography (HPLC) analysis using a Shimadzu LC-20AT (Shimadzu, Kyoto, Japan) with an ultraviolet (UV) detector. The HPLC conditions were as follows: chromatographic column, Shim-PackVP-ODS (250 × 4.6 mm) (Shimadzu, Kyoto, Japan); mobile phase, 1 mL L^−1^ trifluoroacetic acid (A) and methanol (B); column temperature, 30 °C; flow rate, 1.0 mL min^−1^; injection volume, 20 μL. The phenolic compounds were identified by comparing their spectral characteristics with those of the standard compounds at 280 nm. The phenol standards of gallic acid, catechin, corilagin, epicatechin and gallocatechin gallate were purchased from Sigma-Aldrich (Shanghai, China).

### Statistical analysis

The experiments were arranged in a completely randomized design. Experiments for respiration rate were repeated five times, whereas other experiments were repeated three times. Significant differences among different storage times and those among treatments were determined by one-way analysis of variance (ANOVA) using SPSS^®^ version 16.0) (SPSS, Inc., Chicago, IL, USA). Statistical differences were assessed with a significance level of 5 %. The results are presented as the means of replicates ± standard error (SE). The graph was drawn by SigmaPlot 10.0.
